# Supplementary material for: Morphology-Controlled Synthesis of V1.11S2 for Electrocatalytic Hydrogen Evolution Reaction in Acid Media
Source: Molecules. 2022 Nov 18;27(22):8019. doi: 10.3390/molecules27228019 (PMC9695646; doi:10.3390/molecules27228019)
Supplement: Supplementary file 1 [file molecules-27-08019-s001.zip › molecules-2009453-supplementary.pdf]

Support information

Morphology-Controlled Synthesis of $V_{1.11}S_2$ for Electrocatalytic Hydrogen Evolution Reaction in acid media

Qiuyue Chen [a, b, c], Siqi Tian [b], Xiaonan Liu [a, *], Xuguang An [b], Jingxian Zhang [b], Longhan Xu [b], Weitang Yao [b], Qingquan Kong [b, *]

a College of Chemical Engineering, Sichuan University of Science & Engineering, Zigong 643000, China

b School of Mechanical Engineering, Chengdu University, Chengdu 610106, China

c School of Chemical Engineering, Sichuan University, Chengdu 610065, China

Corresponding author: Tel.: +86 28 84616169; fax: +86 28 84616169.

E-mail address: kongqingquan@cdu.edu.cn (Q.Q. Kong); lxn@suse.edu.cn (X. Liu)

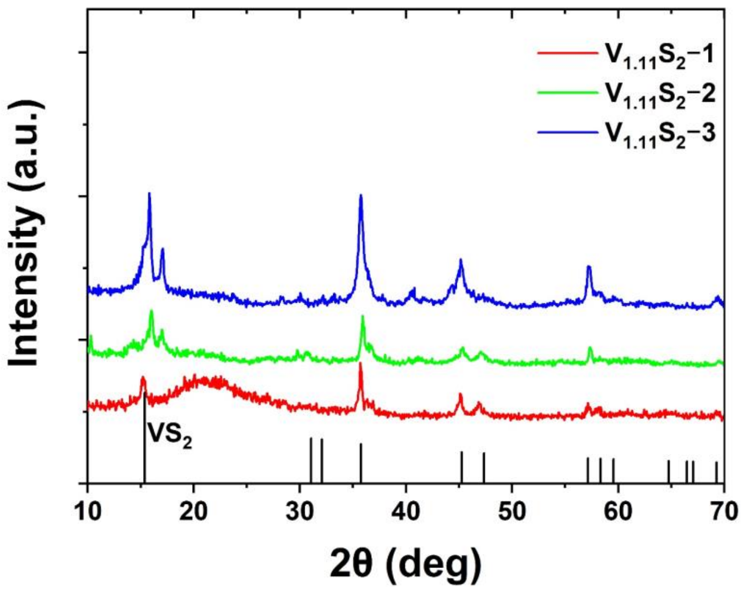

Figure S1 XRD patterns of hydrothermal synthesis precursor powder before annealing.

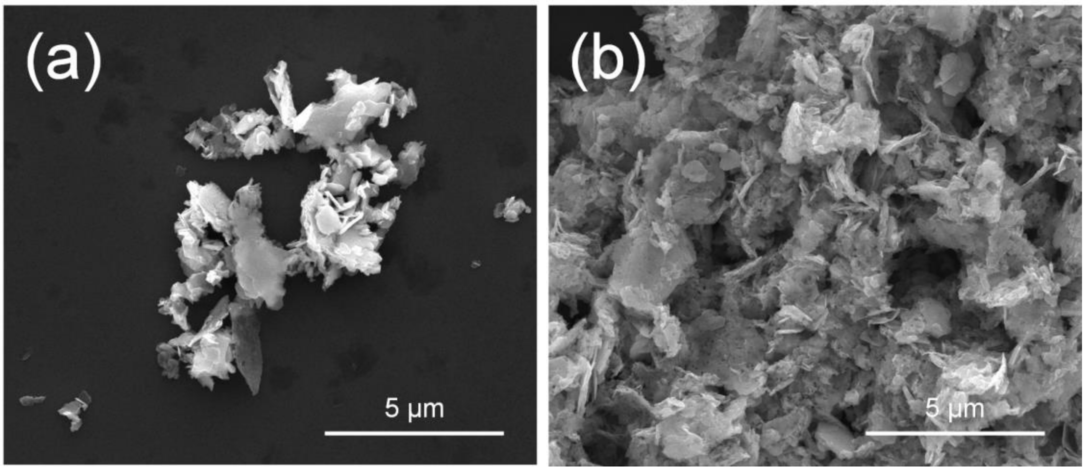

Figure S2 FE-SEM images of (a) $V_{1.11}S_2$-2, and (b) $V_{1.11}S_2$-3 materials.

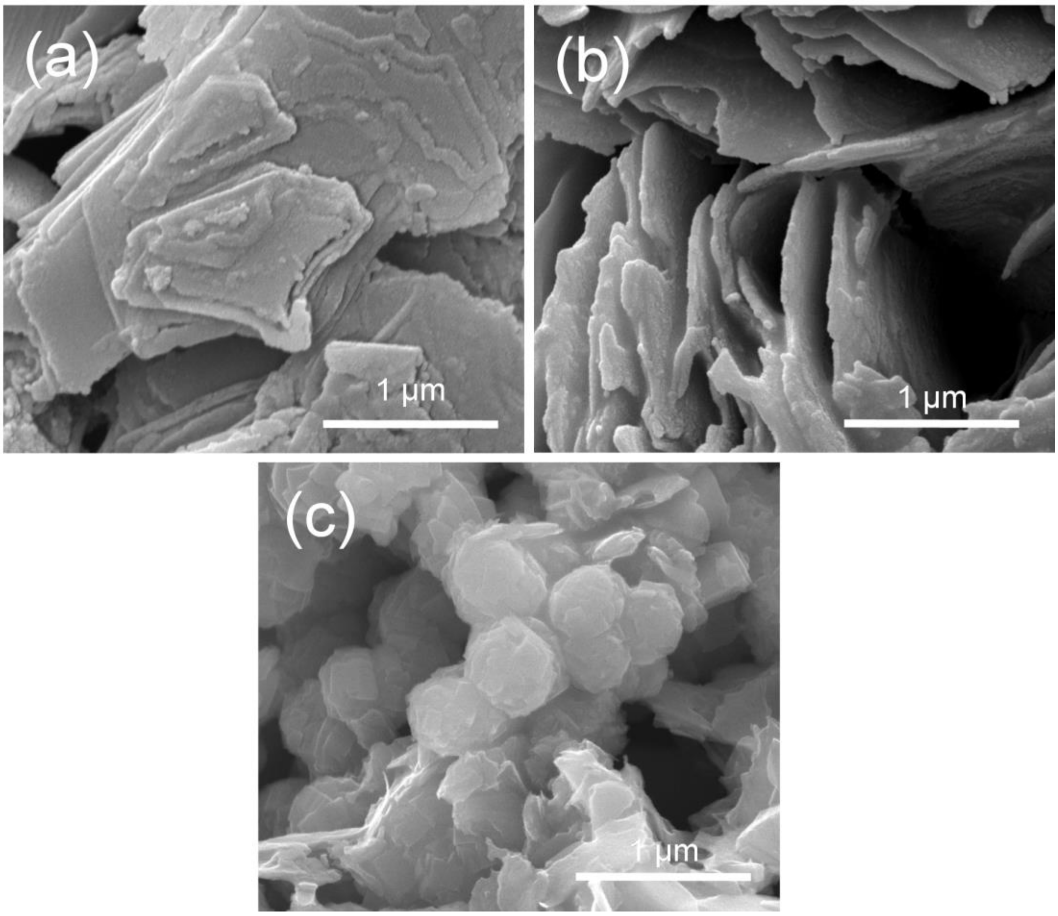

Figure S3 FE-SEM images of precursor powder before annealing, (a) $V_{1.11}S_2$−1, (b) $V_{1.11}S_2$−2, and (c) $V_{1.11}S_2$−3, respectively.

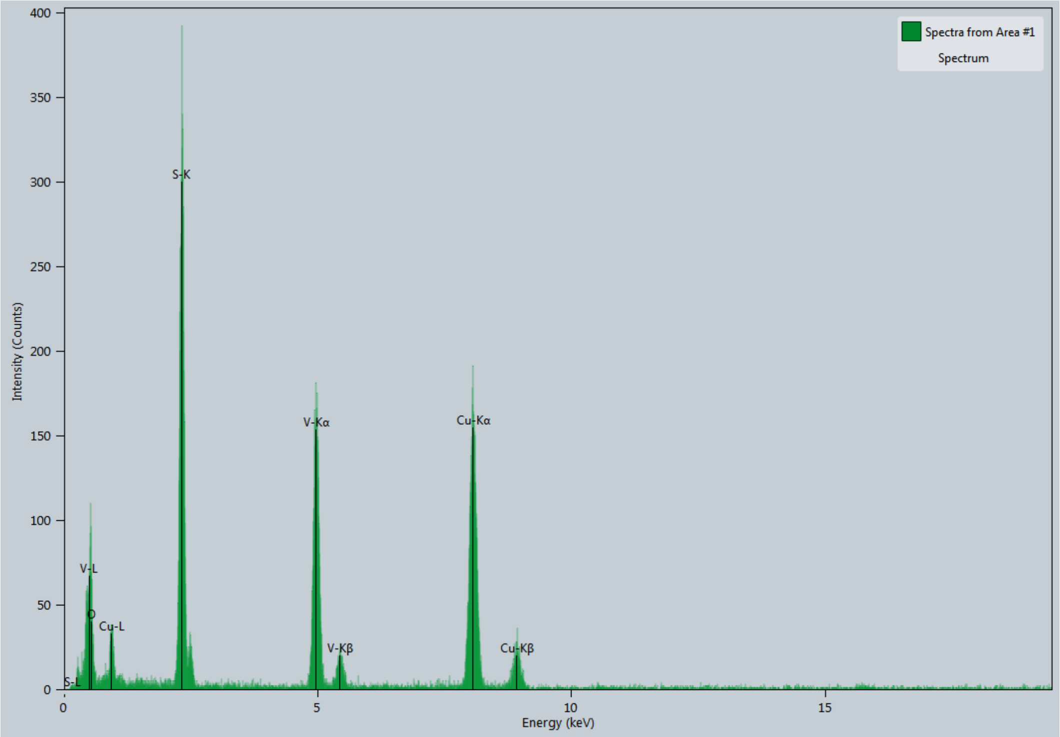

Figure S4 EDS pattern of $V_{1.11}S_2$−1 materials.

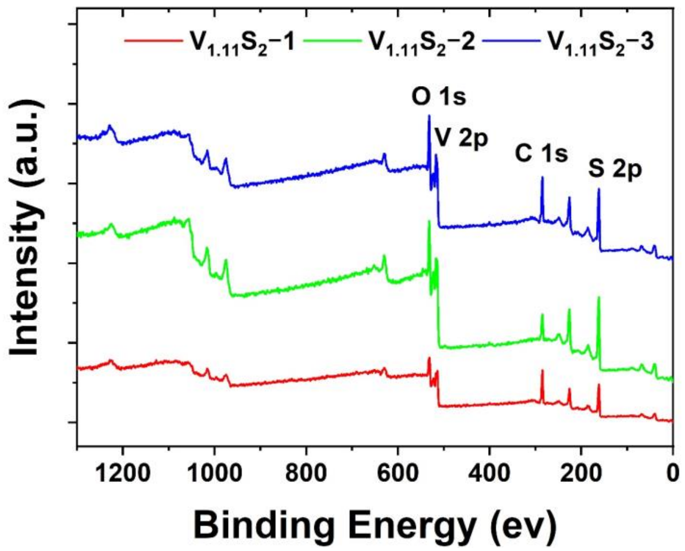

Figure S5 XPS survey spectra of $V_{1.11}S_2$−1, $V_{1.11}S_2$−2, and $V_{1.11}S_2$−3 materials.

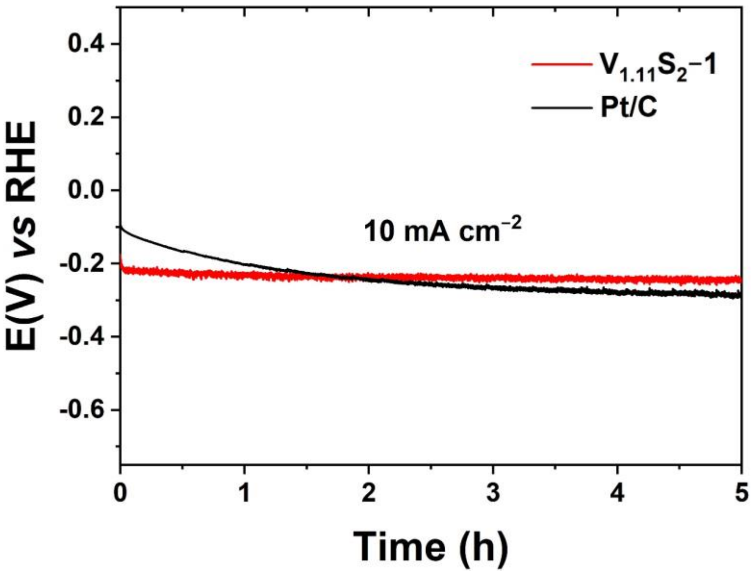

Figure S6. Chronopotentiometry curve of $V_{1.11}S_2$−1 and Pt/C recorded at the current density of 10 mA cm^{-2} for a total duration of 5 h.

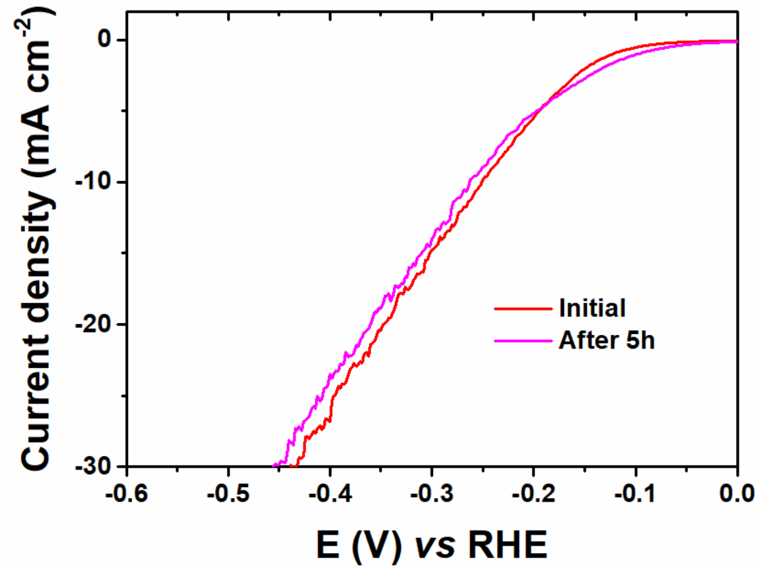

Figure S7. Initial (red line) and after 5 h (purple line) polarization curves of $V_{1.11}S_2$−1 material.

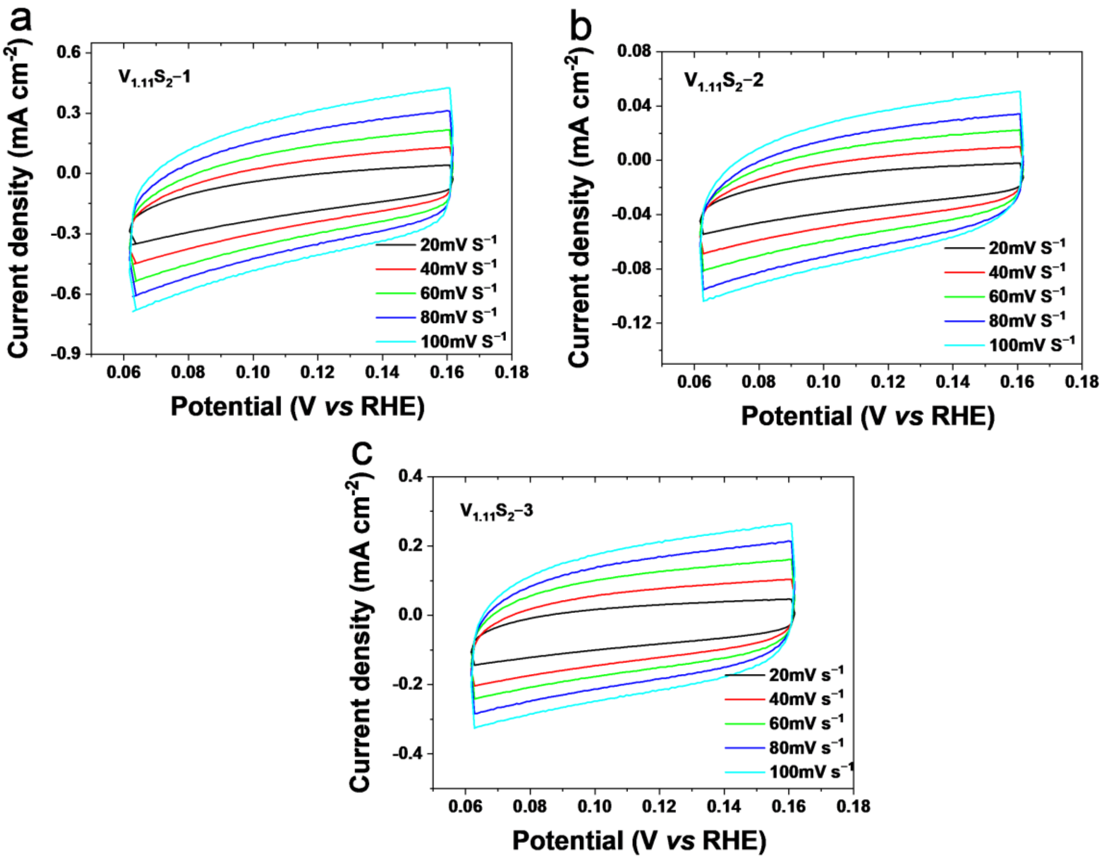

Figure S8. Voltammograms of (a) $V_{1.11}S_2$−1, (b) $V_{1.11}S_2$−2 and (c) $V_{1.11}S_2$−3 at various scan rates of 20, 40, 60, 80 and 100 mV s^{-1}, respectively.

Table S1 The electric double layer (Cdl) capacitance is obtained by fitting CV curve

Catalyst	Cdl	R-Square
$V_{1.11}S_2$−1	3.4	0.9993
$V_{1.11}S_2$−2	0.45	0.9999
$V_{1.11}S_2$−3	1.9	0.9988
